# Bacterial Infection Disrupts the Intestinal Bacterial Community and Facilitates the Enrichment of Pathogenic Bacteria in the Intestines of *Penaeus vannamei*

**DOI:** 10.3390/microorganisms13040864

**Published:** 2025-04-10

**Authors:** Renjun Zhou, Shaoping Weng, Jianguo He

**Affiliations:** 1 State Key Laboratory of Biocontrol/School of Marine Sciences, Sun Yat-sen University, Guangzhou 510275, China; 2 School of Life Sciences/Southern Marine Sciences and Engineering Guangdong Laboratory (Zhuhai)/China-ASEAN Belt and Road Joint Laboratory on Mariculture Technology, Sun Yat-sen University, Guangzhou 510275, China

**Keywords:** *Penaeus vannamei*, bacterial infection, *Vibrio parahaemolyticus*, intestine microbiota, *Ruegeria*

## Abstract

Pathogenic infections can reshape the intestinal microbiota of aquatic animals, thereby impacting their health status. In this study, we aimed to investigate whether *Vibrio parahaemolyticus* infection induces dysbiosis in the intestinal bacterial community of *Penaeus vannamei* and to assess the associated ecological risks. Our findings revealed the deterministic processes in intestinal bacterial community assembly during bacterial infections, indicating that host selection, i.e., host immune response post-infection, has a significant influence on intestinal microbes. More importantly, we found that bacterial infection reshaped the intestinal community by reducing the relative abundance of probiotic *Ruegeria* species (e.g., *R. atlantica*, *R. lacuscaerulensis*, *R. conchae*, *R. profundi*, *R. arenilitoris*, *R. pomeroyi*) and increasing the relative abundance of *Vibrio* species (*V. harveyi*, *V. sinaloensis*, *V. coralliilyticus*, and *V. brasiliensis*). Significant negative correlations were observed between the relative abundance of these *Ruegeria* species and the relative abundance of *Vibrio* species. Moreover, the control *P. vannamei* contained a substantially higher number of keystone species belonging to *Ruegeria* in the bacterial community network, whereas bacterial infection individuals had few or no keystone species belonging to *Ruegeria*, with keystone species belonging to *Vibrio* becoming more prominent. Thus, the significant increase in *Vibrio* species abundance in the *P. vannamei* intestine following bacterial infection was associated with the marked reduction in *Ruegeria* species. Our findings will provide valuable insights into the complex interactions among bacterial infection, intestinal microbiota, and host health, and they provide guidance for the development of probiotics in promoting the healthy culture of *P. vannamei*.

## 1. Introduction

*Penaeus vannamei*, commonly known as the Pacific white shrimp, is the most extensively farmed shrimp species globally, accounting for over 50% of total shrimp production [1]. Currently, the global aquaculture of *P. vannamei* faces a significant threat from bacterial diseases. *Vibrio parahaemolyticus*, one of the most prevalent bacterial pathogens affecting crustaceans, fish, and shellfish, particularly harboring a virulence plasmid encoding PirA/B, can cause acute hepatopancreatic necrosis disease in shrimp [2,3]. This disease can lead to 100% mortality among infected shrimp populations, resulting in substantial economic losses for the shrimp-farming industry [4,5]. Consequently, the prevention and control of vibriosis in shrimp pose a critical challenge for both scientific research and industrial practices.

The intestinal microbiota plays crucial roles in resisting pathogen invasion and maintaining host health [6,7,8,9,10]. Thus, a stable intestinal microbiota is vital for host health, and disruptions to this equilibrium can adversely affect the host [11]. Pathogenic infections can significantly alter the composition of intestinal microbiota in aquatic animals, characterized by reduced community diversity and pronounced shifts in specific bacterial taxa within the host intestines. For instance, high-dose infections with *Vibrio vulnificus* and *V. parahaemolyticus* lead to a marked decrease in the diversity index of the intestinal bacterial community in *Cynoglossus semilaevis* and *Penaeus vannamei* [12,13]. Similarly, viral infections induce significant alterations in the intestinal microbiota of aquatic animals; for example, grass carp reovirus infection in *Ctenopharyngodon idellus* resulted in decreased community diversity and the dysbiosis of intestinal microbiota, leading to a substantial expansion of *Cetobacterium* in the host’s intestine [14]. These findings have substantially advanced our understanding of the relationship between pathogenic infections and intestinal microbiota homeostasis in aquatic animals. However, further research is needed to fully comprehend how pathogenic infections impact host health through disruption of intestinal microbiota.

Multiple studies have provided compelling evidence that the disruption of the intestinal bacterial community can contribute to the *Vibrio* infection and the development of bacterial diseases in *P. vannamei* [15,16,17,18]. Additionally, a study by Zhang et al. [13] highlighted significant alterations in both the composition and function of the intestinal bacterial community following *V. parahaemolyticus* infection. These changes included an increase in the abundance of potential pathogenic bacteria and a decrease in beneficial bacteria, which were closely associated with metabolic disorders of nucleotides, lipids, amino acids, and carbohydrates within the host’s intestine. This research offers novel insights into the pathogenesis of vibriosis induced by the *V. parahaemolyticus* infection in *P. vannamei*. However, the mechanisms by which *V. parahaemolyticus* infection modulates the intestinal microbiota to affect its preventive function against *V. parahaemolyticus* and the subsequent disease progression remain unclear.

A previous study has indicated that the shrimp infection rate following injection was 40.4 times greater than that following immersion by *V. parahaemolyticus* [19]. Research reported signaling events induced by lipopolysaccharide-activated Toll in response to *V. parahaemolyticus* injection in shrimp, and this further induced antimicrobial peptides [20]. The shrimp’s antibacterial activities (PO and T-NOS) and the expression of the antibacterial genes (*proPO*, *ALF*, *Toll*, and *Imd*) and pathogen pattern recognition genes (*LGBP* and *Lec*) increased at first and then decreased after LPS injection [21]. Here, our objective was to investigate whether bacterial infection induces dysbiosis in the intestinal bacterial community of *P. vannamei* and to assess the associated ecological risks. Specifically, we evaluated the changes in bacterial community diversity and compositions within the *P. vannamei* intestines during infection by *V. parahaemolyticus* and lipopolysaccharides. We also focused on identifying probiotics from *P. vannamei* intestines that significantly decrease in response to infections. Our findings will provide valuable insights into the complex interactions among pathogens, intestinal microbiota, and host health and provide guidance for the development of the probiotics of healthy *P. vannamei* culture.

## 2. Materials and Methods

### 2.1. Experimental Design and Sample Collection

We conducted a controlled experiment to address our research questions. In this experiment, shrimp seedlings were free from specific pathogens. All foreign substances, including seawater and feed, were disinfected and tested for pathogens. We further determined that the experimental shrimp were not infected by examining each individual shrimp, ensuring that they met the following criteria: no macroscopic signs of disease; a complete hepatopancreas with clear margins; and a full intestine. During the experiment, the shrimp culture’s water temperature varied with indoor natural temperatures, and salinity was set at 30‰; each aquarium had the same breeding density, with 30 shrimp per aquarium. The shrimps were given commercial feeds 4 times (7:00, 11:00, 15:00, and 20:00) every day (approximately 1 g feed for each aquarium); residual feed and feces in the aquarium were cleaned 90 min after feeding. Initially, *P. vannamei* (8.73 ± 0.66 cm length and 5.14 ± 1.11 g weight) were randomly allocated to aquariums for acclimatization. After three days of acclimatization, the shrimp were divided into three groups: the *V. parahaemolyticus* (Vpa) group, the lipopolysaccharide (LPS) group, and the control group. Each group comprised 30 shrimp. We conducted intramuscular examinations in the experiment. The *V. parahaemolyticus* used in the experiment was isolated from hepatopancreatic necrosis shrimp, harboring a virulence plasmid encoding PirA/B [18]. *V. parahaemolyticus* was cultured in a Luria-Broth medium for more than 12 h and then diluted in PBS to achieve a concentration of approximately 10^3^ CFU per 50 μL. Each shrimp in the Vpa group was injected with 50 μL of this bacterial suspension. Each shrimp in the LPS group was injected with 5 μg of LPS dissolved in 50 μL of PBS, while each shrimp in the control group received 50 μL of PBS alone [22]. Intestinal samples were collected at 72 h post-injection. For each group, five randomly selected shrimp served as individual samples. During sampling, the shrimp surfaces were disinfected using a 70% ethanol solution. The intestines were carefully dissected using sterile scissors, and their contents were gently scraped off. The collected intestinal contents were transferred into 2 mL centrifuge tubes containing 1.5 mL of a PBS buffer. All samples were stored at −80 °C prior to DNA extraction.

### 2.2. The 16S rRNA Sequencings and Bioinformatic Analysis

Intestinal genomic DNA was extracted using the PowerFecal DNA Isolation Kit (Qiagen, Düsseldorf, Germany). The DNA quality control and PCR amplification were performed according to our previous study [23]. Sequencing libraries were constructed as previously described [24]. A qualified library was sequenced on a PacBio Sequel II platform (Pacific Biosciences, Menlo Park, CA, USA) by Biomarker Technologies Co., Ltd. (Beijing, China), generating single-end reads. Raw data (PRJNA1234576) were deposited in the SRA maintained by NCBI. Raw sequences were filtered and demultiplexed using SMRT Link (version 8.0) to obtain circular consensus sequencing (CCS) reads with minPasses ≥ 5 and minPredictedAccuracy ≥ 0.9, which were further processed with Lima (version 1.7.0). The forward and reverse primers on these reads were identified via cutadapt (version 2.7); if the error rate in primer identification exceeded 20%, the sequence was discarded. CCS reads lacking primer sequences were removed, and the remaining CCS reads were filtered based on a length threshold of 1200–1650 bp. Each sample yielded more than 5000 CCS reads. The UCHIME algorithm (V8.1) [25] was used for chimera detection and removal, resulting in a set of clean reads. Sequences with 100% similarity were clustered into amplicon sequence variants (ASVs) using USEARCH (V10.0). Taxonomy annotation was performed based on SILVA SSU NR99 database version 138. Alpha-diversity (the diversity index reached a plateau; see Figure S1a) and beta-diversity were calculated using QIIME2 [26], and a phylogenetic tree was constructed using MEGAN analysis [27].

### 2.3. Statistical Analysis

Venn analysis was conducted to compare the differences in bacterial OTU numbers among the three groups. Student’s *t*-test was used to evaluate the differences in bacterial diversity indices between any two groups. Non-metric multidimensional scaling (NMDS), permutational multivariate analysis of variance (PERMANOVA), and heatmap analyses were performed to assess the dissimilarities in community structure between the groups based on the Bray–Curtis distance [18]. LEfSe analysis was employed to identify bacterial indicators at the genus and species levels in the control, Vpa, and LPS groups. Spearman’s correlation analysis was utilized to examine the relationships between different bacterial taxa. SparCC analysis [28] was applied to assess the complexity of bacterial community interactions within each group at the species level over time, with a significance threshold of |r| > 0.5 and *p* < 0.05. The mean nearest taxon distance measure was then used to determine the processes governing community assembly in *P. vannamei* intestines across the three groups [29,30].

## 3. Results

### 3.1. Differences in Intestinal Bacterial Diversity of the Control, Vpa, and LPS Groups

**Figure 1:**
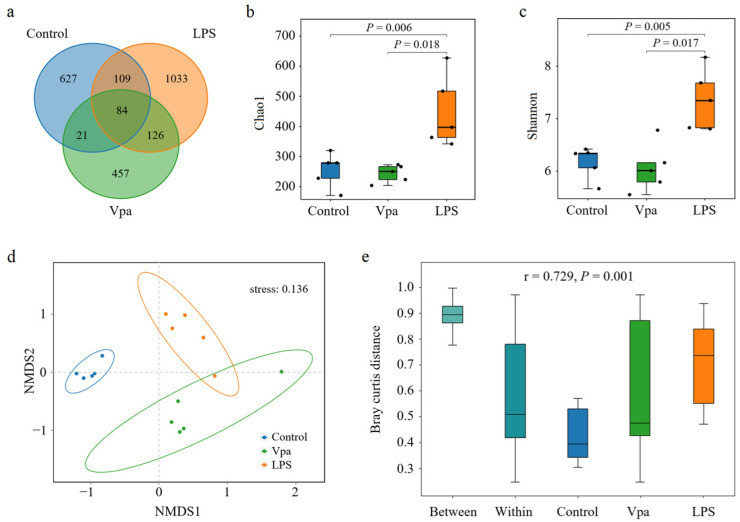
(**a**) Venn analysis results of bacterial ASV numbers in the *Penaeus vannamei* intestine of the control, Vpa, and LPS groups. (**b**,**c**) Comparative analysis of the Chao1 and Shannon indices in the bacterial community within *P. vannamei* intestines among the control, Vpa, and LPS groups. (**d**,**e**) NMDS and PERMANOVA results of community structure within the *P. vannamei* intestines among the control, Vpa, and LPS groups. Vpa: *Vibrio parahaemolyticus*; LPS: lipopolysaccharide.

The number of OTUs in the intestine of the Vpa group showed a slight decrease compared to the control group, whereas the LPS group exhibited a substantial increase relative to both the control and Vpa groups (Figure 1a). Student’s *t*-test results indicated that the Chao1 (Figure 1b) and Shannon (Figure 1c) indices of the Vpa group (243 ± 29 and 6.05 ± 0.46) were numerically lower than those of the control group (255 ± 57, 6.16 ± 0.31), although they were not statistically significant (*p* > 0.05). In contrast, the LPS group had significantly (*p* = 0.006 and = 0.018; *p* = 0.005 and = 0.017) higher Chao1 (449 ± 120) and Shannon (7.36 ± 0.58) indices compared to both the control group and the Vpa group. Regarding β-diversity, the NMDS analysis (stress = 0.136) (Figure 1d) and heatmap results (Figure S1b) demonstrated significant variations in community structures within *P. vannamei* intestines across all pairwise comparisons, as supported by PERMANOVA analysis (r = 0.729, *p* = 0.001) (Figure 1e). Notably, the intestinal bacterial community structure of *P. vannamei* in the Vpa and LPS groups (87.40 ± 4.97% distance) was more similar to each other than to that of the control group (90.07 ± 4.35% distance, *p* < 0.05. Figure 1d,e and Figure S1b). These findings suggested that bacterial infection significantly altered the diversity of the intestinal bacterial communities in *P. vannamei*.

### 3.2. Differences in Bacterial Composition of the Control, Vpa and LPS Groups

**Figure 2:**
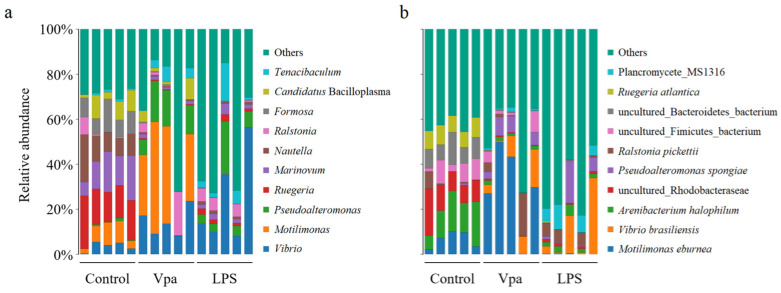
Relative abundances of top ten bacterial taxa within the *P. vannamei* intestines in the control, Vpa, and LPS groups at the (**a**) genus and (**b**) species levels. Vpa: *Vibrio parahaemolyticus*; LPS: lipopolysaccharide.

**Figure 3:**
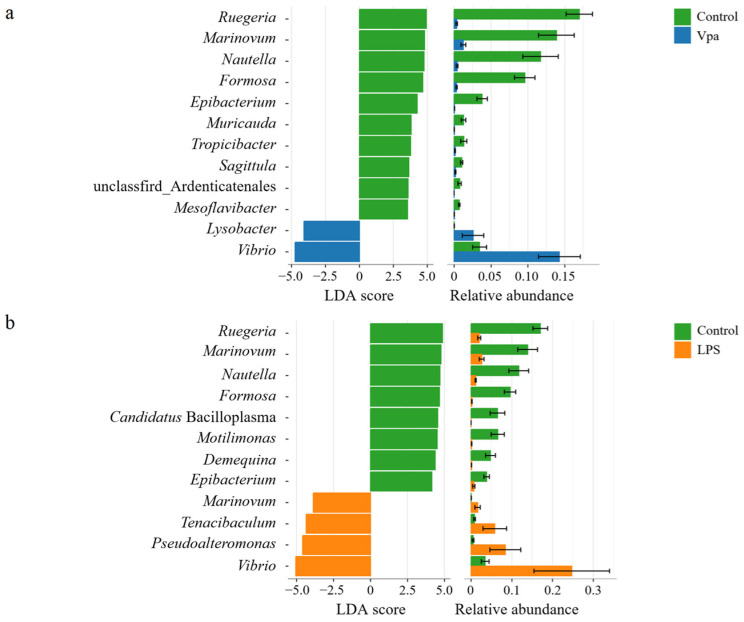
(**a**) LEfSe results of bacterial indicator genera within the *P. vannamei* intestines in the control and Vpa groups. (**b**) LEfSe results of bacterial indicator genera between the control and LPS groups. Vpa: *Vibrio parahaemolyticus*; LPS: lipopolysaccharide.

**Figure 4:**
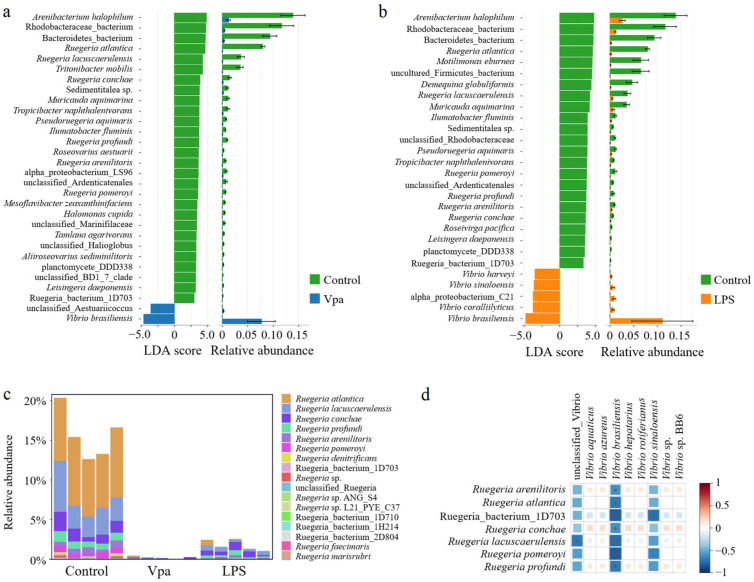
(**a**) LEfSe results of the bacterial indicator species within *P. vannamei* intestines in the control and Vpa LPS groups and (**b**) between the control and LPS groups. (**c**) Relative abundances of *Ruegeria* species within the *P. vannamei* intestines in the control, Vpa, and LPS groups. (**d**) Spearman’s correlation analysis was utilized to examine the relationships between *Ruegeria* species and *Vibrio* species; * means *p* < 0.05. Vpa: *Vibrio parahaemolyticus*; LPS: lipopolysaccharide.

Obviously, alterations in the bacterial taxonomic composition of host intestines were observed in the Vpa and LPS groups compared to the control group. Specifically, the relative abundances of *Ruegeria*, *Marinovum*, and *Nautella* decreased in the Vpa and LPS groups, while *Vibrio* and *Pseudoalteromonas* showed the opposite trends (Figure 2a). At the species level, *R. atlantica* abundance decreased in the Vpa and LPS groups, but *Vibrio brasiliensis* and *Pseudoalteromonas spongiae* increased (Figure 2b). LEfSe analysis revealed that *Ruegeria*, *Marinovum*, *Nautella*, and *Formosa* were key biomarkers of the control *P. vannamei*, while *Lysobacter* and *Vibrio* were dominant in the Vpa group (Figure 3a), and *Vibrio*, *Marinovum*, *Tenacibaculum*, and *Pseudoalteromonas* were prominent in the LPS group (Figure 3b). At the species level, 28 indicator species were identified in the control group compared to the Vpa group, 7 of which belonged to *Ruegeria* (*R. atlantica*, *R. lacuscaerulensis*, *R. conchae*, *R. profundi*, *R. arenilitoris*, *R. pomeroyi*, and Rugeria_bacterium_1D703); only 2 indicator species were found in the Vpa group, with 1 being *V. brasiliensis* (Figure 4a). Similarly, 23 indicator species were identified in the control group compared to the LPS group, with 7 belonging to *Ruegeria* (also *R. atlantica*, *R. lacuscaerulensis*, *R. pomeroyi*, *R. profundi*, *R. arenilitoris*, *R. conchae*, and Rugeria_bacterium_1D703); 5 indicator species were found in the LPS group, 4 of which belonged to *Vibrio* (*V. harveyi*, *V. sinaloensis*, *V. coralliilyticus*, and *V. brasiliensis*) (Figure 4b). Therefore, bacterial infection reshaped the intestinal community by reducing the relative abundance of *Ruegeria* and increasing the relative abundance of *Vibrio*. We further observed that the relative abundance of 17 *Ruegeria* species was markedly reduced in both the Vpa and LPS groups, with some species, particularly *R. atlantica* and *R. lacuscaerulensis*, decreasing to nearly 0% in the Vpa group (Figure 4c). Spearman’s correlation analysis revealed significant (*p* < 0.05) negative correlations between the relative abundance of all seven *Ruegeria* indicator species and *V. brasiliensis* (Figure 4d). Additionally, there were significant (*p* < 0.05) negative correlations between *R. lacuscaerulensis* and unclassified_Vibrio, as well as between Rugeria_bacterium_1D703 and *V. sinaloensis* (Figure 4d). These suggested that the significant increase in Vibrio abundance in *P. vannamei* intestines following bacterial infection was associated with the marked reduction in *Ruegeria*.

### 3.3. Differences in Bacterial Co-Association Networks and Ecological Processes on the Intestinal Community Assembly Among the Control, Vpa, and LPS Groups

**Figure 5:**
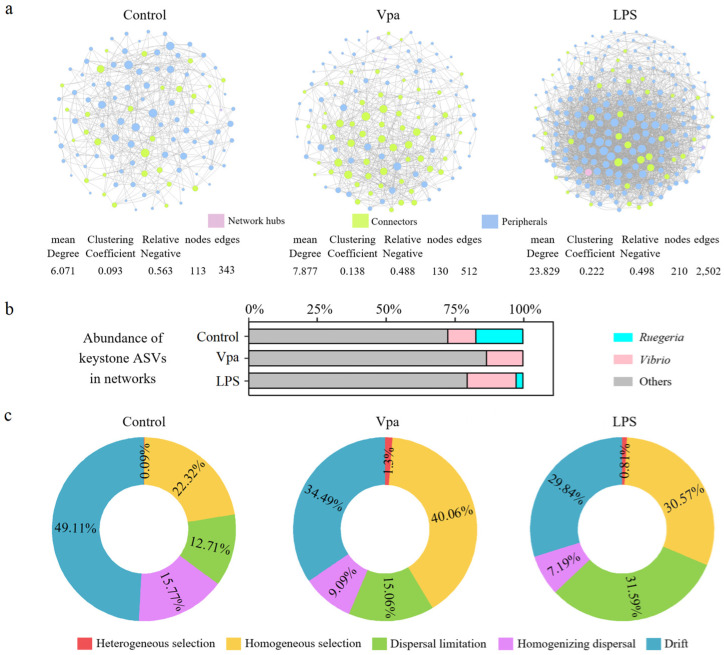
(**a**) Distinctions in co-association networks of the bacterial community within the *P. vannamei* intestines were observed among the control, Vpa, and LPS groups. (**b**) A substantially higher number of keystone species belonging to the *Ruegeria* species in the control group, but more *Vibrio* keystone species in the Vpa and LPS groups. (**c**) Differences in ecological processes affecting the bacterial community assembly in the intestine of *P. vannamei* among the control, Vpa, and LPS groups. Vpa: *Vibrio parahaemolyticus*; LPS: lipopolysaccharide.

We utilized SparCC analysis to examine the impact of bacterial infection on the co-association networks of bacterial species in *P. vannamei* intestines. Our results demonstrated that both the number of nodes and number of edges in the bacterial networks were significantly higher in the Vpa (130 nodes and 512 edges) and LPS (210 nodes and 2502 edges) groups compared to the control group (113 nodes and 343 edges) (Figure 5a). Notably, the control group contained a substantially higher number of keystone species belonging to the *Ruegeria* species, whereas the Vpa and LPS groups had few or no keystone species from this genus, with the *Vibrio* species becoming more prominent (Figure 5b). Consequently, under the conditions of bacterial infection, there was a marked increase in the complexity of the bacterial networks within *P. vannamei* intestines, which may be associated with the significant reduction in *Ruegeria* and concurrent rise in *Vibrio*. We further quantified the relative contributions of major ecological processes to evaluate bacterial assembly in *P. vannamei* intestines across three groups (Figure 5c). Overall, approximately half of the observed variation was attributed to stochastic processes: drift (49.11%, 34.49%, and 29.84%), dispersal limitation (12.71%, 15.06%, and 31.59%), and homogenizing dispersal (15.77%, 9.09%, and 7.19%) in the control, Vpa, and LPS groups, respectively. This indicates that stochastic factors play a significant role in shaping the bacterial community in *P. vannamei* intestines. However, deterministic processes such as heterogeneous selection and homogeneous selection were notably higher in the Vpa (1.30% and 40.06%) and LPS (0.81% and 30.57%) groups compared to the control group (0.09% and 22.32%). This suggested that deterministic factors have a greater influence on intestinal bacterial community assembly in Vpa and LPS *P. vannamei*. Consequently, bacterial infection might result in strong selective pressure on the intestinal bacterial community, potentially leading to a decline in the native dominant bacteria *Ruegeria* in *P. vannamei* intestines.

## 4. Discussion

Host response to pathogen infection modulates the intestinal microbial community composition, which in turn adversely affects the host’s health. This study specifically investigated the effects of bacterial infection on the intestinal microbiota of *P. vannamei* and its interaction with host health. It is evident that pathogen infection can elicit an extensive immune response against host microbes, as demonstrated in previous studies showing the upregulation of immune-related genes in *P. vannamei* [2,31] under the conditions of *V. parahaemolyticus* infection. These immune responses further interact with commensal bacteria in hosts, thereby altering the bacterial community composition in the host intestine. This observation is supported by a pronounced deterministic process in intestinal community assembly under bacterial infection, indicating that the host immune response, i.e., host selection post-infection, has a significant influence on the intestinal microbes. More precisely, our study found that the intestinal bacterial community of infected individuals exhibited obvious dysbiosis compared to the control *P. vannamei*, characterized by a marked decrease in *Ruegeria* (e.g., *R. atlantica*, *R. lacuscaerulensis*, *R. conchae*, *R. profundi*, *R. arenilitoris*, *R. pomeroyi*) abundance and significant enrichment of pathogens (e.g., *V. harveyi*, *V. brasiliensis*). The excessive expansion of pathogens is likely associated with intestinal microbiota dysbiosis, characterized by a disruption in the microecological balance and a diminished host defense against pathogen invasion [32,33].

*Ruegeria* is a symbiotic bacterium in the intestine of *P. vannamei* that plays a crucial role in enhancing the host’s resistance to bacterial infections and maintaining intestinal homeostasis [34]. Our results demonstrated that bacterial infection reshaped the intestinal community by reducing the abundance of *Ruegeria* species and increasing the abundance of *Vibrio* species, and significant negative correlations between the abundance of all seven *Ruegeria* indicator species and *Vibrio* species were observed. Moreover, the control *P. vannamei* contained a substantially higher number of keystone species belonging to the *Ruegeria* species, whereas bacterial infection individuals had few or no keystone species from the *Ruegeria* genus, with *Vibrio* species becoming more prominent. Thus, the significant increase in *Vibrio* species abundance in *P. vannamei* intestines following bacterial infection was associated with the marked reduction in *Ruegeria* species. In fact, several studies have confirmed that the enrichment of *Ruegeria* in the *P. vannamei* intestine enhances the host’s antagonistic response to *V. parahaemolyticus* [35], thereby reducing the likelihood of bacterial digestive tract diseases, including white feces syndrome [2,3]. Therefore, the secondary expansion of opportunistic pathogens such as *Vibrio* in the *P. vannamei* intestine following bacterial infection may be associated with a significant reduction in *Ruegeria* populations. Since LPS could trigger the host’s antimicrobial peptide pathway [20], we hypothesized that *Ruegeria* might be more susceptible to antimicrobial peptides induced by LPS injection in comparison to *Vibrio* as Gram-negative bacteria [36] according to our results. Consequently, bacterial infection can disrupt the intestinal bacterial community of *P. vannamei*, particularly leading to the depletion of native intestinal bacteria such as *Ruegeria* species, thereby promoting the proliferation of bacterial opportunistic pathogens and increasing the risk of bacterial disease in the host.

In addition, *R. arenilitoris*, an indigenous intestinal bacterium of *P. vannamei*, holds significant potential as a probiotic in aquaculture, which aids in enhancing resistance against *Vibrio* infections. A study meticulously formulated a probiotic mixture comprising four strains, *Ruegeria lacuscaerulensis*, *Nioella nitratireducens*, *Bacillus subtilis*, and *Streptomyces euryhalinus*, in a specific ratio; moreover, dietary supplementation with these probiotic blends promoted beneficial intestinal bacteria, enhanced short-chain fatty acid production, boosted taurine metabolism potential, and improved bacterial network stability while it reduced the turnover rate and average variation degree of intestinal community, thereby reinforcing both ecological and mechanical barriers against *Vibrio* pathogens in *P. vannamei* [37]. Thus, *Ruegeria* species may exert an antagonistic effect against Vibrio pathogens in the intestine of *P. vannamei* through the aforementioned functions, yet the precise anti-*Vibrio* mechanism requires further investigation.

## 5. Conclusions

In conclusion, our study specifically investigated the effects of bacterial infection on the intestinal bacterial community of *P. vannamei* and its interaction with the host’s health. We mainly found that bacterial infection reshaped the intestinal bacterial community by reducing the relative abundance of probiotic *Ruegeria* species and increasing the relative abundance of pathogenic Vibrio species, and the significant increase in *Vibrio* was associated with the marked reduction in *Ruegeria*. Our findings enhance the comprehension of the complex interactions among bacterial infection, intestinal microbiota, and host health, and they provide guidance for the development of probiotics for the healthy culture of *P. vannamei*.

## Data Availability

The original contributions presented in this study are included in the article/Supplementary Material. Further inquiries can be directed to the corresponding author.

## Supplementary Materials

The following supporting information can be downloaded at: https://www.mdpi.com/article/10.3390/microorganisms13040864/s1, Figure S1: (a) The α-diversity indices in all samples were calculated using rarefaction curves at the ASV level at a sequencing depth of 3885 with 1000 iterations, where the indices had stabilized. (b) Heatmap results of bacterial community structure within the *P. vannamei* intestines among the control, Vpa, and LPS groups.
