# Supplementary figures and images for: Bacterial Infection Disrupts the Intestinal Bacterial Community and Facilitates the Enrichment of Pathogenic Bacteria in the Intestines of Penaeus vannamei
Source: Microorganisms. 2025 Apr 10;13(4):864. doi: 10.3390/microorganisms13040864 (PMC12029295; doi:10.3390/microorganisms13040864)

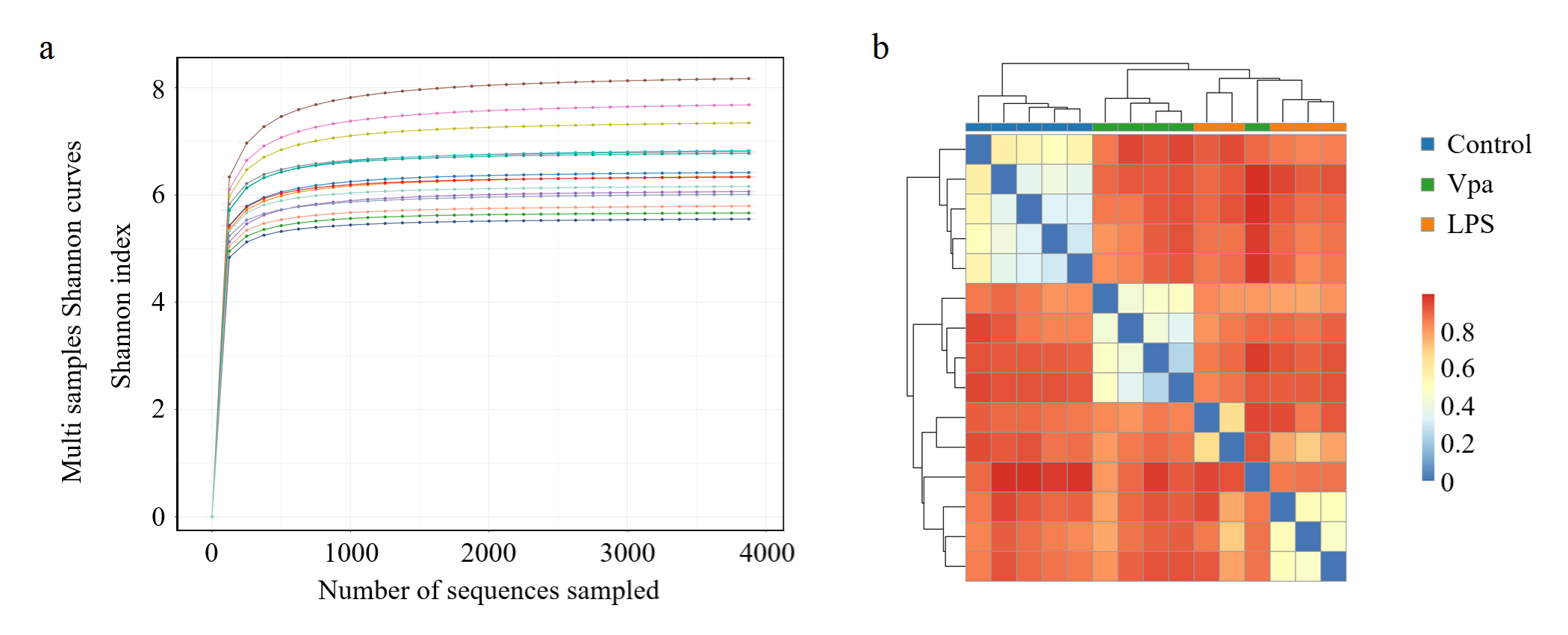

Supplement: Supplementary file 1 [file microorganisms-13-00864-s001.zip › Figure S1.tif]
